# Contrasting Retromer with a Newly Described Retriever in *Arabidopsis thaliana*

**DOI:** 10.3390/plants13172470

**Published:** 2024-09-04

**Authors:** Connor D. Lewis, Mary L. Tierney

**Affiliations:** Department of Plant Biology, University of Vermont, Burlington, VT 05405, USA; connor.lewis@uvm.edu

**Keywords:** retromer, retriever, VPS35A, VPS26C, VPS29, CCDC22, CCDC93, root hair, endosomal trafficking

## Abstract

The tight regulation of protein composition within the plasma membranes of plant cells is crucial for the proper development of plants and for their ability to respond to a changing environment. Upon being endocytosed, integral membrane proteins can be secreted, sorted into multivesicular bodies/late endosomes, and degraded in the lytic vacuole, or recycled back to the plasma membrane to continue functioning. The evolutionarily conserved retromer complex has attracted the interest of plant cell biologists for over a decade as it has emerged as a key regulator of the trafficking of endocytosed integral plasma membrane proteins. Recently, a related recycling complex that shares a subunit with retromer was described in metazoan species. Named “retriever”, homologs to the proteins that comprise this new recycling complex and its accessory proteins are found within plant lineages. Initial experiments indicate that there is conservation of function between metazoan and plant retriever proteins, suggesting that it is prudent to re-evaluate the available plant retromer data with the added potential of a plant retriever complex.

## 1. Introduction

The endomembrane system provides the evolutionarily conserved network of vesicular trafficking pathways that underlie the physiology of eukaryotic cells. Its major components are the nuclear envelope, endoplasmic reticulum (ER), Golgi apparatus, trans-Golgi network (TGN), plasma membrane, endosomes, multi-vesicular bodies/late endosomes (MVBs/LEs), and vacuole. The coordinated function of these cellular compartments is responsible for the establishment and maintenance of the distinct collections of proteins found within these organelles, as well as the proteins and lipids found on the plasma membrane and within the extracellular space. In plants, the endomembrane system contributes to numerous biological processes, including phototropism and gravitropism [1], the polarized growth of root hairs [2] and pollen tubes [3], nutrient acquisition [4] seed development [5], and innate immunity [6]. As they are integrated into such diverse and important processes, disruptions to vesicular trafficking pathways can have severe pleiotropic effects including infertility or death in plants [7], or disease pathologies such as Parkinson’s disease in humans (*Homo sapiens*) [8].

Two important regulators of membrane traffic, retromer and retriever, will be the focus of this review. While retromer was first investigated in plants over twenty years ago [9], retriever was only recently identified in a human cell culture [10] and has not been extensively studied in plants to date. However, phylogenetic data suggest the maintenance of retriever components in plant lineages, while molecular studies suggest the conservation of function between human and plant retriever components. Furthermore, VPS29 (vacuolar protein sorting 29) is a subunit shared by both the core retromer and retriever, indicating that re-examination of the plant retromer literature is warranted in the context of the discovery of retriever.

## 2. Fates of Endocytosed Cargo

As sessile organisms, plants need to be able to dynamically regulate the proteins found on their plasma membranes to adapt to changing environmental conditions. Upon being endocytosed, integral membrane proteins can be secreted, degraded in the lytic vacuole, or rescued from the degradative pathway and recycled back to the plasma membrane to continue functioning [11]. Degradation relies on the evolutionarily conserved multi-subunit endosomal sorting complex required for transport (ESCRT) machinery. In eukaryotes, ESCRT-0, ESCRT-I, ESCRT-II, ESCRT-III, and the VPS4 complexes are sequentially recruited to endosomal membranes, where they cluster ubiquitinated cargo proteins and facilitate the inward budding and ultimately the fission of the membrane into the luminal space of endosomes, which creates the characteristic intraluminal vesicles (ILVs) of MVBs/LEs [12]. The ultimate fate of an MVB depends upon the membrane with which it fuses. MVB fusion with the plasma membrane will release the ILVs as exosomes, while fusion with the tonoplast will result in the degradation of ILVs and the cargo proteins that stud their membranes [13]. Of note, plant genomes do not encode ESCRT-0 proteins, but the plant-specific protein FREE1 (FYVE DOMAIN PROTEIN REQUIRED FOR ENDOSOMAL SORTING 1) has been shown to perform comparable functions as it works with ESCRT-I complexes to initiate ILV formation [14]. See Gao et al. for an excellent review of ESCRT-mediated processes in plants [15].

The TGNs of plant cells are dynamic organelles where multiple trafficking routes converge. Newly synthesized secretory traffic and vacuolar proteins mature through the TGN, while they also receive endocytic traffic from the plasma membrane [16]. This is in contrast to mammalian cells, in which endocytic traffic converges at early endosomes (EE), distinct organelles from which plasma membrane protein degradation (via ESCRT-mediated processes) or recycling can take place [17]. It is hypothesized that maintaining a pool of active plasma membrane proteins within endosomes is important biologically because the opportunity to dynamically recycle or degrade proteins as needed allows cells to respond to changing environmental conditions quickly. This strategy may also be less energy-intensive than synthesizing new proteins whenever they are needed. Several classes of proteins that function on the plasma membrane have been shown to be recycled from the plant TGN/EE back to the plasma membrane, including auxin transporters [18], leucine-rich repeat receptor-like kinases [19,20], and nutrient transporters [21,22]. The molecular interactions that facilitate the recycling of plasma membrane proteins in plants are not as well defined as those involved in trafficking cargo to the lytic vacuole, but multiple studies have implicated the evolutionarily conserved retromer complex [23].

## 3. Retromer in Yeast and Mammals

Genetic screens designed to define proteins essential for vacuolar protein sorting in *Saccharomyces cerevisiae* resulted in the identification of retromer, a protein complex necessary for the retrograde trafficking of the yeast transmembrane receptor Vps10p [24,25]. Vps10p interacts with its cargo, vacuolar hydrolase carboxypeptidase Y (CPY), at the Golgi and sorts CPY into vesicles. Vesicles containing CPY are transported to the endosome, which fuses with the vacuole upon maturation, at which point CPY is delivered to the vacuolar lumen. Retromer enables single Vps10p proteins to be involved in multiple rounds of CPY trafficking, by facilitating the retrograde transport of Vps10p from endosomes back to the Golgi. The lack of a functional retromer complex results in the mis-localization of Vps10 and causes CPY to be secreted from cells [24].

In yeast, retromer functions as a pentamer composed of two subcomplexes. A smaller, sorting nexin (SNX) complex composed of Vps5p and Vps17p anchors retromer to membranes, while the larger, core retromer complex consisting of Vps35p, Vps29p, and Vps26p was found to interact with cargo proteins, including vacuolar hydrolases [25]. The characteristic p40 phox-homology (PX) domain of the SNXs can bind phosphatidylinositol 3-phosphate (PI3P) and anchor the core retromer complex to endosomal membranes, while their Bin-Amphiphysin-Rvs (BAR) domains deform membranes into tubulovesicular shapes which help create retrograde transport vesicles [26,27]. Since its initial discovery, retromer has been extensively studied and is now considered to play important roles in cellular trafficking pathways in all eukaryotes [28]. There are, however, some important differences between retromer function in yeast and mammals.

In mammals, there has been diversification of the sorting nexin family to 33 members, all of which contain the PX domain and potentially additional functional domains. There are two Vps5p homologs, SNX1 and SNX2, and three Vps17p homologs, SNX5, SNX6, and SNX32, that represent the SNX-BAR proteins in mammals [29]. Like their yeast counterparts, these proteins form heterodimers; however, they only transiently interact with the core retromer trimer of VPS35, VPS29, and VPS26 [30] (referred to as retromer from here on).

The vacuole of yeast is analogous to the lysosome of mammalian cells, and both require receptors to transport hydrolases to their lumens to break down cellular waste products. One such receptor in mammals is the cation-independent mannose 6-phosphate receptor (CI-MPR), which performs an analogous function to Vps10p and is recycled from endosomes back to the TGN [31]. Recent investigations using super-resolved stimulated emission depletion (STED) microscopy suggest that VPS35 and the SNX-BAR protein SNX1 occupy distinct domains on endosomes in HeLa cells [32], and that the retrograde transport of CI-MPR can happen through distinct retromer-mediated, SNX-BAR-mediated, and Retromer/SNX-BAR-independent pathways [33]. This suggests that unlike in yeast, retromer function does not rely on SNX-BAR proteins, and in fact SNX-BAR proteins can carry out recycling functions without retromer. Interestingly, in addition to the vacuolar trafficking pathways it is involved in, retromer is also involved in an EE-to-plasma membrane recycling pathway. Together with SNX27 (a non-BAR domain containing sorting nexin) acting as a cargo adaptor, retromer is responsible for the correct surface expression of over 100 transmembrane proteins [34,35].

## 4. Plasma Membrane Protein Recycling by Retromer and Retriever

Recently a heterotrimeric protein complex with structural similarity to retromer was identified in human cell culture and determined to be central to an early endosome-to-plasma membrane recycling pathway [10]. Named “retriever”, the complex is composed of VPS35L (formerly C16orf62), VPS29, and VPS26C (formerly DSCR3). To perform their recycling functions, both retromer and retriever need to associate with endosomal membranes and interact with cargo proteins; however, they utilize different proteins to perform these functions. To localize to the retrieval subdomains of endosomes, retromer requires SNX3 and GTP-loaded Rab7a, while retriever requires the COMMD/CCDC22/CCDC93 (CCC) and Wiskott–Aldrich syndrome protein and SCAR homologue (WASH) complexes [10,36]. Loss of CCDC22 or CCDC93 (Coiled-Coil Domain-Containing) prevents retriever from associating with the WASH complex on endosomes and carrying out its recycling functions [10].

Non-BAR domain-containing sorting nexins act as cargo adaptors on endosomes for both retromer and retriever. SNX17 is capable of recognizing NxPY/NxxY-motif-containing cargoes and coupling these cargoes to retriever to facilitate their recycling to the plasma membrane. The association between SNX17 and retriever in humans appears to be dependent upon VPS26C, as SNX17 and retriever do not coprecipitate in VPS26C-KO cell lines [10]. SNX27 associates with retromer and helps it recycle cargoes with PDZ-binding motifs [37]. Recent analyses revealed that SNX17 and SNX27 utilize their respective FERM domains in dramatically different ways, and these differences explain how the two proteins facilitate the recycling of different cargoes despite their structural similarities [38]. McNally et al. (2017) identified proteins that were present at the surface of SNX17-KO cells at significantly lower levels than in control cells [10]. Among these proteins were established SNX17 cargoes as well as integral membrane proteins required for cell adhesion, numerous nutrient transporters, and signaling receptors. A proportion of the proteins identified by this approach overlapped with proteins identified using the same approach but in SNX27-depleted cells, suggesting an overlap between retromer and retriever cargoes.

## 5. Plant Retromer and Retriever

**Figure 1:**
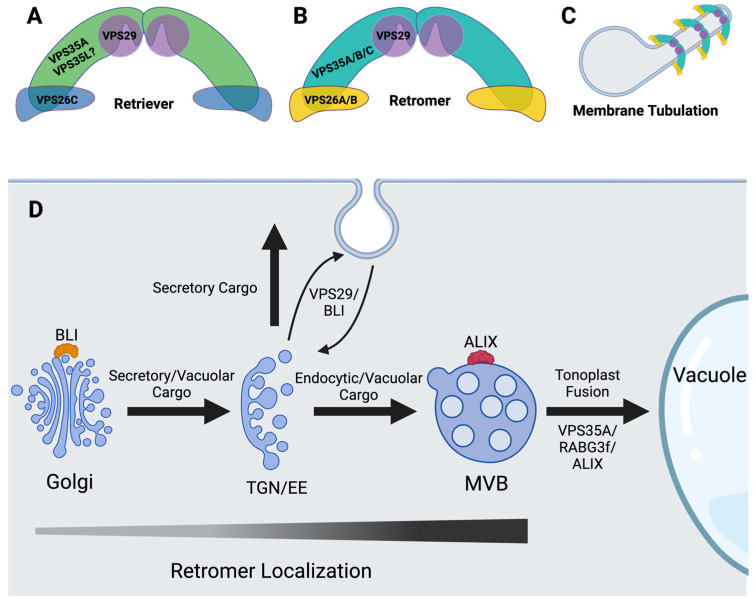
An overview model of retriever and retromer structures, functions, and localizations in plants. (**A**) Retriever and (**B**) retromer are both described as trimers that oligomerize into higher-order structures. Two trimers come together to form an arch-like structure with the largest subunit, VPS35, being connected to VPS26 homologs at the base and VPS29 at the top. Retromer and retriever are differentiated by the VPS26 member present; VPS26C is part of retriever and VPS26A and VPS26B are part of retromer. Furthermore, in plants, a putative retriever complex has been described as containing VPS35A, while retromer complexes can comprise VPS35A, VPS35B, or VPS35C. A homolog to VPS35L of *Homo sapiens* has recently been identified but has not been studied in plants to date. We have placed a ? next to VPS35L in the model because there is no data showing an interaction between VPS35L and either VPS29 or VPS26C in plants to date. (**C**) When bound to membranes, retriever and retromer create a tubular–vesicular structure and provide a scaffold-like matrix for protein–protein interactions to take place. (**D**) Retromer is central to vesicular trafficking in plant cells, having been demonstrated to play a role the recycling of PIN proteins to the plasma membrane in collaboration with BLISTER (BLI), which localizes predominantly to the Golgi but also the TGN and MVB. Furthermore, retromer is essential for MVB/tonoplast fusion, a process in which ALIX and RABG3f also play major roles due to their abilities to regulate retromer’s localization to MVBs. Retromer localization represents a gradient of VPS29 and VPS26A colocalization with SYP32 (Golgi), VHA-a1 (TGN), and Rha1 (MVB) markers, as reported by Hu et al., 2022 [39].

The genetic analysis of retromer function in plants implicates it in many aspects of development. The core retromer complex consists of a heterotrimer of VPS35, VPS29, and VPS26. In Arabidopsis (*Arabidopsis thaliana*), VPS35 is encoded by three paralogs and VPS26 is encoded by 2 paralogs, while VPS29 is a single-copy gene (Figure 1).

An analysis of the VPS35 gene family showed that while the growth of *vps35a*, *vps35b,* and *vps35c* single mutants was very similar to that of the wild type, the *vps35bvps35c* double mutant and the *vps35avps35bvps35c* triple mutant were retarded in their growth in a manner similar to the *vps29* null mutant (see below, [7]). In contrast, the *vps35avps35c* double mutant grew very similarly to wild-type plants. The *vps35bvps35c* double mutant exhibited early leaf senescence phenotypes and smaller protein storage vacuoles, and these mutants secreted storage proteins into the extracellular space [7]. These results implicate VPS35B in trafficking pathways to the storage vacuole and suggest that some combinations of the core retromer are not redundant in function. The analysis of the *vps35Avps35bvps35c* triple mutant also implicated core retromer function in seed and embryo development. Many of the embryos were misshaped or exhibited lethality in the triple mutant.

An analysis of *pat3* (protein affecting trafficking), which corresponds to VPS35A, showed an accumulation of the plasma membrane proteins PIN1, PIN2, PIP2, and AUX 1 in internal PVC-like compartments as well as aberrant PVC morphology when plants were grown under nutrient-poor conditions [40]. Similar phenotypes were observed with *vps29* mutants [40]. This study provides evidence for VPS35A and VPS29 function in maintaining PVC morphology and supports a model in which VPS35A plays the major role in trafficking cargo to the lytic vacuole.

Trafficking of cargo to the lytic vacuole starts in the ER. SNX1 and SNX2 function to promote the movement of soluble proteins from the ER to the TGN as well as in the export of vacuolar sorting receptors (VSRs) from the ER [41]. Once at the TGN, VSR family members are separated from their cargo and are trafficked back to the ER through either a retromer- or clathrin-mediated process, and movement of cargo from the TGN to the vacuole is considered receptor-independent, occurring by maturation [42,43]. The function of VSR’s on the MVB membrane is not clear. It is possible that their presence on the MVB membrane reflects a pathway for their degradation in the lytic vacuole or that they are in the process of being recycled back to the ER or Golgi. However, more experimentation will be needed to address this question. SNX1 has also been shown to bind to CLASP1 to mediate the trafficking of PIN proteins to the lytic vacuole for degradation or for the recycling of proteins to the plasma membrane, and this pathway is regulated by gibberellic acid in Arabidopsis [44].

An analysis of *vps29* alleles in Arabidopsis and in maize (*Zea mays*) support the role of the core retromer complex in trafficking proteins to the storage vacuole in developing seeds [5,45]. However, the downregulation of VPS29 in Arabidopsis has severe effects on overall plant growth that are linked to cell polarity and organ initiation in plants [23]. The seedling shoots and roots of *vps29* alleles are shorter than in the wild type, their growth is agravitropic, and rosette and silique development is dramatically altered when compared to wild-type seedlings. VPS29 was found to be necessary both for normal SNX1 endosome morphology as well as PIN1 and PIN2 recycling in seedling roots. These data support a model in which VPS29 is required for the recycling of PIN1 and PIN2 from SNX1 endosomes to the plasma membrane [23]. As PIN1 and PIN2 are auxin efflux transporters in plants, their altered recycling in *vps29* mutants is likely to affect auxin homeostasis during plant growth, contributing to many of the developmental phenotypes observed in this retromer mutant. It should also be noted that VPS29 is also a component of the core retriever (see below) in plants. Therefore, a simultaneous reduction in the retromer and retriever pathways may contribute to some of the developmental phenotypes associated with the growth of *vps29* plants.

Three VPS26 genes (*VPS26A*, *VPS26B*, and *VPS26C*) are expressed in Arabidopsis, and single *vps26a* and *vps26b* mutants have few phenotypes. However, the *vps26avps26b* double mutant is severely compromised in many aspects of growth, suggesting that VPS26A and VPS26B may have many redundant functions [46]. The seedling growth of *vps26avps26b* plants resembles the *vps29* mutants in Arabidopsis as they show altered cotyledon development and are defective in root growth, and their rosettes are much smaller than those observed in wild-type plants. These results suggest that loss of retromer function by eliminating the expression of either VPS26A and VPS26B or VPS29 results in pleiotropic consequences for growth in plants.

## 6. Retromer-Binding Proteins

Recently, several additional core retromer-interacting proteins have been identified, and their functions, with respect to retromer, have been characterized. BLISTER (BLI), a protein found specifically in plant lineages, has been shown to bind all three paralogs of VPS35 in Arabidopsis and to VPS29 and co-localizes with, but has not been shown to bind, the VPS26 paralogs [47]. *bli* mutants disrupt membrane recruitment of the core retromer complex, which disrupts the recycling of endocytosed PIN proteins [47]. This further supports a model in which VPS29 participates in a TGN/EE-to-plasma membrane recycling pathway that regulates PIN localization. BLI localizes mainly to the Golgi but partially to the TGN and, to a lesser extent, MVBs. In addition to its role in a TGN/EE-to-plasma membrane recycling pathway, BLI also influences the trafficking of soluble vacuolar proteins. This is similar to other mutants in which retromer function is compromised, including apoptosis-linked gene-2 interacting protein X (ALIX).

ALIX, a protein previously described as involved in the ESCRT-mediated degradation of membrane proteins and in MVB compartment biogenesis, has been shown to co-localize and physically interact with VPS26A, VPS26B, and VPS29 in plants. ALIX is required for the recruitment of VPS26A and VPS29 to the MVB [39], and these interactions are important for trafficking soluble proteins to the vacuole. With the loss of ALIX, there is the subsequent mis-localization of vacuolar sorting receptors (VSRs) to the plasma membrane, perhaps due to retromer being unable to perform its function of VSR recycling. The importance of ALIX and retromer is underscored by a genetic analysis of *alix* mutants in combination with either *vps26avps26b* double mutants or with *vps29*, which demonstrate essential functions for these complexes during gametophyte and embryo development.

Hu et al. [39] also performed a robust in vivo analysis of retromer localization. By crossing VPS29-GFP- and VPS26A-GFP-expressing lines to lines expressing organelle markers fused to mCherry, they were able to determine the amount of co-localization between retromer and the Golgi, TGN, and MVB. The results were similar for VPS29 and VPS26A in both embryo and root epidermal cells and revealed that the most co-localization was seen with MVB components (roughly 70%), but some co-localization was also observed with the TGN (about 25%) and the Golgi (about 15%). Of note, these results are dependent upon the markers that the group used to define each organelle, which were SYP32, VHA-a1, and Rha1 for the Golgi, TGN, and MVB, respectively. It would be interesting to perform similar experiments with the remaining retromer/retriever components: VPS35A/B/C and VPS26B/C. Such experiments could help tease apart the subtle functional differences between the paralogs that have been observed in other studies [7,48].

To facilitate MVB/tonoplast fusion, retromer participates in an evolutionarily conserved RAB5/RAB7 (ras-associated binding) cascade [49]. Briefly, upon maturation from the TGN, MVBs are recognized by the presence of the RAB5 GTPase RABF2b/ARA7. RABF2b remains on the MVB membrane until the MON1(SAND1)-CZZ1AB complex inactivates it by performing its function as a RAB5 guanosine triphosphate-activating protein (GTPase). Simultaneously, MON1(SAND1)-CZZ1AB can act as a guanine nucleotide exchange factor (GEF) and activate the RAB7 protein RABG3f [50]. The switch of MVBs from a RABF2b- to a RABG3f-positive state allows for a new suite of proteins to be recruited to MVB membranes, including VPS35A-containing retromer complexes, the membrane-tethering homotypic fusion and protein-sorting (HOPS) complex, and the SNAREs SYP22 and VAMP713 [50,51]. These changes give MVBs the competency to fuse with the tonoplast, thereby delivering the contents of the MVBs to the vacuole.

## 7. Is There a Retriever Complex in Plants?

A phylogenetic analysis of genes encoding the core retromer subunits in eukaryotes identified a subclade of *VPS26* genes that were distinct from *VPS26A* and *VPS26B* [29]. These genes were first identified as *DSCR3* genes and included orthologs from both animal and plant genomes. A subsequent analysis of retriever complexes in humans showed that *DSCR3* sequences were represented by the VPS26C subunit of the core retriever complex [10]. A *VPS26C* ortholog in Arabidopsis has been shown to be essential for the polarized growth of root hairs [2]. A similar phenotype was observed for *vps29* and *vps35a* mutants, but null mutants for other *VPS35* and *VPS26* gene family members exhibited normal root hair growth. Bimolecular fluorescence complementation studies showed that VPS26C interacts with VPS35A but not VPS35B or VPS35C. Interestingly, both the Arabidopsis and human *VPS26C* sequences were shown to complement the *vps26c* root hair phenotype. A phylogenetic analysis of VPS26C sequences in plants showed that it is widely distributed among plant genomes but is absent from grasses. Thus, the identification of a VPS35A, VPS29, and VPS26C complex supports the function of a core retriever complex in plants.

*CCDC22* and *CCDC93* are single-copy genes in Arabidopsis; however, homologs of COMMD proteins are not found among seed plant genomes. In humans, the CCC (COMMD, CCDC22, CCDC93) complex physically interacts with retriever, and structural analyses of retriever suggests that the CCC complex forms a bridge between the core retriever and the WASH complex [10,52]. This couples retriever to an activator of Arp2/3-dependent actin polymerization. Genes encoding the COMMD and WASH proteins are absent from seed plant genomes; therefore, it remains an open question as to which proteins coordinate CCDC22 and CCDC93 function in plants. A genetic analysis of *ccdc22* and *ccdc93* mutant alleles in Arabidopsis showed that they are defective both in root hair and root growth and that these phenotypes can be complemented with *CCDD22-* and *CCDC93-RFP* fusions under the transcriptional control of their endogenous promoters [53]. A phylogenetic analysis of *CCDC22* and *CCDC93* homologs in plants showed that these genes share an evolutionary history with *VPS26C*.

While the evidence suggesting a retriever complex in plants is mounting, further studies are needed to demonstrate the basis of the root hair phenotypes observed in the *vps26c, ccdc22*, and *ccdc93* mutants. Disruptions to endosomal trafficking pathways have been posited as an explanation for these phenotypes because polarized growth relies heavily on the coordinated delivery and retrieval of plasma membrane and cell wall materials to the tips of cells undergoing polarized growth. However, experimental data placing VPS26C squarely within the endomembrane system in plants are lacking. Furthermore, physical interactions between the core retriever and possible retriever-interacting proteins, such as CCDC22 and CCDC93, have not been demonstrated in plants to date. Experimental systems that will identify binding partners for these proteins, such as yeast two-hybrid assays, in planta immunoprecipitation analysis, or proximity-labeling experiments, would help address these limitations, confirm whether the constitution of retriever is conserved between plants and humans, and identify components of the endomembrane system that may regulate and/or interact with VPS26C, CCDC22, and/or CCDC93 in plants.

## 8. Genetic Interactions between VTI11- and VTI13-Dependent Pathways to the Lytic Vacuole and Retromer/Retriever Function in Plants

An analysis of a *vps26cvti13* double mutant indicates that *vps26c* is a genetic suppressor of the polarized growth and cell wall organization phenotype of *vti13* [2]. VTI13 is a v-SNARE that localizes to early endosomes and the tonoplast membrane [54]. Similar suppression of the *vti13* root hair growth phenotype can be observed in double mutants between *vti13* and *ccdc93* [53]. These types of interactions are not limited to VTI13. VTI11’s functions in trafficking cargo to the lytic vacuole and mutations in this SNARE result in a shoot agravitropic phenotype [55]. A reverse genetic screen of *vti11* resulted in the identification of *vps26a*, *vps35a*, and *vps29* as genetic suppressors of this shoot agravitropic phenotype [48]. In contrast, *vps26b*, *vps35b*, and *vps35c* were not suppressors of the agravitropic phenotype. Together, these studies support a genetic interaction between a VTI-dependent trafficking pathway to the lytic vacuole and endosomal trafficking pathways involving a retromer and/or a retriever core complex. In addition, they demonstrate that the specific combination of proteins within the core retromer/retriever complex dictates nuanced differences in cellular function.

## 9. Future Directions

Moving forward, there are still many open questions concerning the molecular players involved in plasma membrane recycling, the complex of associated proteins required for retromer and retriever function, and the cargo that is trafficked by retromer and retriever in plants. Much of what we know about plasma membrane protein recycling has been gleaned by following the dynamics of a few distinct proteins. Experimental approaches designed to identify (1) additional cargoes of recycling complexes and (2) proteins that regulate these processes would expand our understanding of the role of retromer and retriever in controlling developmental processes in plants. Recent studies on the retriever complex in humans, using proximity labeling, showed that the core retriever, CCDC22, CCDC93, and COMMD proteins play a role in the binding of specific cargo in vivo [52]. These studies not only supported the role of retriever in known intracellular trafficking pathways, but also revealed its function in regulating cellular processes that had not previously been associated with retriever. Similar approaches are likely to be productive in plants. Complementary studies would include a proteomic analysis of plasma membrane proteins incorrectly localized in the wild type and retromer, retriever, and *snx* mutants as a method for identifying additional cargo proteins specific for retromer- and retriever-dependent pathways, as well as amino acid motifs in cargo proteins that are required for recycling in plants.

While many single mutants of core retromer and retriever proteins are not dramatically altered in terms of their growth and development, the use of suppressor screens has been productive in identifying cellular pathways in which single *VPS26* and *VPS35* family members play an essential role in plants [2,6,48,53]. The continued use of suppressor screens is likely to be an important tool for dissecting the version(s) of retriever and retromer complexes that control the trafficking of specific cargoes required for cellular responses to developmental and environmental signals. It is interesting to note that *VPS29* is the only single-copy gene shared by both the retriever and retromer core complexes. The severe developmental phenotype exhibited by this mutant is likely due to the combined loss of both core retromer and retriever function in plants and underscores the importance of retrograde trafficking pathways in controlling development.

Future questions aimed at characterizing retromer and retriever function in plants will span most aspects of plant development, but will include the following:

1. Is retriever involved in the recycling of plasma membrane proteins in plants and what are its cargoes?
2. BLISTER and ALIX associate with retromer in Arabidopsis. What other proteins are associated with core retromer and retriever complexes in plants to modify/regulate their function?
3. Do CCDC22 and CCDC93 associate with retriever alone or are they also involved in retromer function?
4. Do CCDC22 and CCDC93 interact with other protein complexes in plants to compensate for the loss of COMMD proteins in seed plants?
5. Are specific versions of the VPS35/29/26 complex required for cell-type-specific endosomal trafficking in plants?

## Data Availability

No new data was created as part of this work.

## Abbreviations

ALIX	Apoptosis-linked gene-2-interacting protein X
BAR	Bin-Amphiphysin-Rvs
BLI	BLISTER
CCDC	Coiled-Coil Domain-Containing
CI-MPR	Cation-independent mannose 6-phosphate receptor
CLASP1	Cytoplasmic linker-associated protein 1
COMMD	Commander
CPY	Carboxypeptidase Y
DSCR3	Down syndrome critical region 3
EE	Early endosome
ER	Endoplasmic reticulum
ESCRT	Endosomal sorting complex required for transport
FREE1	FYVE DOMAIN PROTEIN REQUIRED FOR ENDOSOMAL SORTING 1
GEF	Guanine nucleotide exchange factor
GTPase	Guanosine triphosphate-activating protein
ILV	Intraluminal vesicles
LE	Late endosome
MBV	Multivesicular body
PI3P	Phosphatidylinositol 3-phosphate
PIN1	Pin-formed
RAB	ras-associated binding
RFP	Red fluorescent protein
SCAR	Suppressor of cAMP receptor
SNX	Sorting nexin
TGN	*trans*-Golgi network
VPS	Vacuolar protein sorting
VSR	Vacuolar sorting receptor
VTI	VPS10 interacting
WASH	Wiskott–Aldrich syndrome
